# Fetal Alcohol Spectrum Disorder Awareness, Knowledge, and Practice Among Australian Occupational Therapists: A Cross‐Sectional Survey

**DOI:** 10.1111/1440-1630.70118

**Published:** 2026-08-18

**Authors:** Laine Chilman, Daniel Meloncelli, Alison Houghton, Asmita Mudholkar, Catherine Hilly

**Affiliations:** ^1^ School of Health University of the Sunshine Coast Queensland Australia; ^2^ QCIF Digital Research Ltd Australia; ^3^ Central Queensland Centre for Rural and Remote Health James Cook University Emerald Queensland Australia; ^4^ La Trobe Rural Health School La Trobe University Bendigo Victoria Australia; ^5^ School of Allied Health, Australian Catholic University and Healthy Brain and Mind Research Centre Australian Catholic University Australia

**Keywords:** allied health, clinical reasoning, fetal alcohol effects, fetal alcohol syndrome, PAE

## Abstract

**Introduction:**

Fetal Alcohol Spectrum Disorder (FASD) describes a spectrum of birth defects and developmental disabilities resulting from prenatal alcohol exposure. With a prevalence rate of 3.64%, developing an FASD‐informed occupational therapy workforce is essential across all practice contexts. Since the Australian diagnostic guidelines were introduced in 2016 and updated in 2025, awareness has grown. This study aimed to explore Australian occupational therapists' awareness, knowledge, and practice regarding FASD.

**Method:**

A cross‐sectional survey of 90 occupational therapists assessed knowledge, awareness, and confidence regarding FASD via a 22‐item online questionnaire. Chi‐square tests examined associations between key variables; effect sizes were estimated using Cramér's V. Inductive content analysis was performed on open‐ended responses.

**Consumer and Community Involvement:**

The survey was designed for occupational therapists practising in Australia to obtain information on current practice contexts and was piloted by occupational therapists working with the FASD community.

**Results:**

Despite 93.3% agreeing FASD knowledge is important, confidence and preparedness were markedly low. Only 26.7% had completed professional development, 37.8% reported easy access to reliable information, and 66.7% were unprepared to discuss FASD concerns. Professional development was strongly associated with confidence in recognising FASD (66.7% vs. 12.1%, P < 0.001, V = 0.517) and confidence working with adolescents and children. Clinical experience showed a categorical threshold effect: 40.7% with experience reported confidence in recognising FASD versus 0% without. Access to reliable information was a significant predictor of preparedness (58.8% vs. 17.9%, P = 0.008). Qualitative findings highlighted an absence of FASD‐specific guidance, with occupational therapists drawing on frameworks and knowledge from other diagnoses.

**Conclusion:**

This study indicates a need for occupational therapy‐specific FASD research and practice guidance. Access to reliable information and professional development is critical to building workforce confidence and preparedness in FASD recognition and management.

## PLAIN LANGUAGE SUMMARY

Fetal Alcohol Spectrum Disorder (FASD) is a complex condition caused by alcohol exposure before birth. It can be difficult to identify and has high costs for individuals, families, and the health‐care system. In Australia, diagnostic criteria were introduced in 2016 and updated in 2025. Occupational therapists play an important role in supporting people with FASD through referrals, assessments, and ongoing care—yet no previous research had examined what Australian occupational therapists know about FASD or how prepared they feel to work with this group.

We surveyed 90 occupational therapists across Australia about their knowledge, confidence, and practice regarding FASD. Despite nearly all participants agreeing that FASD knowledge is important in their work, most reported low confidence and felt unprepared to discuss FASD concerns with clients. Only around one in four had completed any formal FASD training, and fewer than four in 10 found reliable information easy to access.

Our findings show that occupational therapists who had completed professional development or had direct clinical experience with FASD were considerably more confident in recognising and supporting people with the condition. Access to reliable information was also strongly linked to feeling prepared to have FASD‐related conversations with clients.

A key limitation is that the sample may over‐represent therapists already interested in FASD, meaning gaps in knowledge and confidence could be even greater across the broader workforce.

These findings highlight the need for occupational therapy–specific practice guidance and improved access to existing FASD resources and training.

**Key Points for Occupational Therapy**

- Significant gaps exist in Australian occupational therapists' knowledge and awareness of FASD.
- Professional development and clinical experience improve confidence and preparedness when working with individuals with FASD.
- Occupational therapy‐specific research and practice guidance is needed to better support individuals with FASD.

## 1 INTRODUCTION

Fetal Alcohol Spectrum Disorder (FASD) describes a spectrum of birth defects and developmental disabilities, caused by prenatal alcohol exposure (PAE) (Popova et al., 2023; Reid, 2018). Individuals with FASD have a diffuse acquired brain injury affecting multiple brain regions and functions, including executive functioning, adaptive behaviour, memory, motor, language, and emotional regulation (Australian Guidelines Development Group, 2024; Bower & Elliott, 2016; Shelton et al., 2018). These difficulties are lifelong, commonly chronic, poorly diagnosed, and costly to the health‐care system (Finlay‐Jones et al., 2020). The Australian prevalence rate is 3.64%, equating to one child per classroom (Tsang et al., 2025). The estimated mean cost per child with FASD, based on US, Canadian, Swedish and New Zealand studies, was $US22,810 (Finlay‐Jones et al., 2020). FASD is the leading cause of preventable non‐genetic disability in Australia, yet a third of Australians are unaware that drinking alcohol during pregnancy can cause FASD, and 23% believe one to two drinks while pregnant is safe (Foundation for Alcohol Research and Education, 2020). FASD is a lifelong and often hidden disability, associated with secondary outcomes, including addiction, justice system involvement, mental health difficulties, and adverse physical health challenges (Finlay‐Jones et al., 2020; Passmore et al., 2018; Petrenko et al., 2014; Popova et al., 2023).

In Australia, FASD diagnosis is governed by the Australian Guidelines for Assessment and Diagnosis of FASD (Australian Guidelines Development Group, 2024), updated in 2025 from the Australian Guide to the Diagnosis of FASD (Bower & Elliott, 2016). Diagnostic criteria require severe neurodevelopmental impairments in at least three domains, with substantial impact on their daily living, assessed via a multidisciplinary approach. Given the high prevalence rate, the 2025 guideline updates, and a rapid review of FASD research priorities in Australia (Finlay‐Jones et al., 2020), there is potential for increased FASD awareness nationally. This is further supported by the Australian National FASD Strategic Action Plan 2018–2028 (Australian Government Department of Health, 2018) and access pathways through the National Disability Insurance Scheme and Medicare Complex Neurodevelopmental Disorders. Occupational therapists encounter people with FASD in all areas of practice, including medical, mental health, and disability. Occupational therapists can contribute to FASD diagnoses by referring individuals with PAE for screening and by providing neurodevelopmental assessments of motor performance, adaptive behaviour, and functional impact (Australian Guidelines Development Group, 2024). Post‐diagnosis, occupational therapists play a key role in ongoing support, informed by clients' neurological profile, environmental factors, and occupational demands.

Individuals with FASD may experience challenges across daily activities, including sleeping, dressing, eating, toileting, family relationships, classroom participation, and peer socialisation (Skorka et al., 2020). Occupational therapists support participation across home, school, work, and community environments. Developing an FASD‐informed occupational therapy workforce in Australia is therefore essential, regardless of practice context.

Research conducted in the United States, Canada, Europe, and New Zealand has reviewed the knowledge and awareness of health‐care professionals in relation to FASD, including but not limited to occupational therapists (Bagley, 2019; Brimacombe et al., 2008; Chu et al., 2023; Garavelis et al., 2024; Kerimofski et al., 2024; Maclean et al., 2014; McCormack et al., 2022; McLachlan et al., 2021; Rudeen et al., 2007; Wedding et al., 2007). However, no known research has been published to date that surveys Australian occupational therapists' awareness, knowledge, or FASD practice. Whilst Skorka et al. (2025) have explored the role and scope of occupational therapy practice for children with FASD, this paper is explicitly a theoretical/conceptual piece rather than an empirical study, drawing on existing literature and clinical case examples rather than collecting data from practising occupational therapists.

### 1.1 Awareness

This current study defines awareness as occupational therapists' confidence to identify and support individuals with FASD or suspected FASD and their families, their preparedness to discuss concerns and initiate referrals, and their recognition of potential FASD within their caseload (Australian Guidelines Development Group, 2024; Lesinskienė et al., 2024; Popova et al., 2023). Two US studies have investigated health professionals' awareness of FASD. Brimacombe et al. (2008) found that an educational talk improved health‐care professionals' FASD awareness and practice scores, whereas Birch et al. (2016) found that targeted training improved participants' confidence in identifying FASD‐related deficits. An aware occupational therapy workforce is critical to supporting individuals and families both pre‐ and post‐diagnosis.

### 1.2 Knowledge

FASD knowledge is defined in this study as occupational therapists' understanding of FASD, their access to reliable evidence‐based information and resources, and their engagement in ongoing learning (Birch et al., 2016; Brimacombe et al., 2008; Evans et al., 2024). It also includes reflective awareness of one's own preparedness and educational needs—recognising not only what is known but also how practitioners identify gaps and pursue ongoing learning (Birch et al., 2016). Multiple studies have found that health and education professionals perceive themselves as lacking FASD knowledge and FASD‐specific resources (Chu et al., 2023; Evans et al., 2024; McCormack et al., 2022; McLachlan et al., 2021; Stubbs et al., 2024). This knowledge gap undermines confidence and capacity to support individuals with FASD and their families and is a significant barrier to accessing FASD services (Chu et al., 2023; Stubbs et al., 2024). Clinicians have reported valuing access to additional FASD training, support, and resources to strengthen professional reasoning and practice (Chamberlain et al., 2017; McLachlan et al., 2021; Rudeen et al., 2007).

### 1.3 Practice

Occupational therapy practice is defined here as the actions occupational therapists take when supporting individuals with FASD or suspected FASD and their families, including the models, frameworks, and philosophies guiding their assessment and intervention (Egan & Restall, 2022). This study examined the characteristics of occupational therapists' FASD‐related practice, including age of clients seen, duration of support, and the clinical reasoning informing their approach. Understanding what occupational therapists do and what informs their clinical reasoning may identify areas for targeted FASD‐specific training and resources, addressing knowledge gaps and strengthening practice outcomes (Rudeen et al., 2007).

### 1.4 Aim

This study aims to explore Australian occupational therapists' perceived awareness, knowledge and practices used to support individuals with FASD or suspected FASD and their families.

## 2 METHODS

### 2.1 Survey Procedure

This cross‐sectional descriptive survey was administered online via QualtricsXM between May and June 2024. A sample of 90 Australian occupational therapists completed the survey in a single session via a web link, addressing their knowledge, awareness, and practices regarding FASD. The 22‐item instrument comprised six domains: demographics (gender, years of practice, country of training, workplace context); awareness and knowledge of FASD (importance of knowledge, information‐seeking, ease of sourcing reliable information); clinical experience (referrals for screening, prior support of FASD clients, suspected undiagnosed FASD); confidence and preparedness (confidence in accessing reliable sources, recognising FASD, and discussing concerns); age‐group‐specific confidence (children, adolescents, adults, and families); and resource awareness and utilisation (10 FASD‐related resources). Ethics approval was obtained from the Human Research Ethics Committee at The University of the Sunshine Coast.

### 2.2 Survey Design

The survey was informed by current FASD literature (Brimacombe et al., 2008; Chu et al., 2023; McCormack et al., 2022; Rudeen et al., 2007) and developed using a tailored design method (Dillman et al., 2014), comprising four sections: demographics, knowledge, awareness, and practice. Knowledge items addressed how therapists first became aware of FASD, whether they sought further information, what resources they accessed, and completion of FASD‐related professional development (PD). Awareness items assessed confidence in working with and recognising FASD and preparedness to discuss concerns and initiate referrals. The practice section was completed only by participants who had supported an individual with FASD in their occupational therapy role, as it captured day‐to‐day practice characteristics and included two numerical items (age at first contact, average caseload duration) and two open‐ended items exploring practice models and reasoning, and desired knowledge and resources.

The survey incorporated quantitative items (binary, Likert‐type, and multiple‐selection formats) and two open‐ended qualitative items inviting participants to describe their clinical reasoning and identify knowledge and resource needs in their own words. The instrument was not formally validated; however, items were developed to align with existing frameworks of FASD knowledge and occupational therapy practice. The survey was piloted with two occupational therapists familiar with the topic, with feedback informing minor revisions prior to final review by the research team. It took approximately 15–20 minutes to complete and was accessed via a secure link, with informed consent obtained through the initial item.

### 2.3 Sampling

Eligible participants were qualified occupational therapists based in Australia, registered with AHPRA (2024), and willing to complete an anonymous online survey; this approach constitutes purposive convenience sampling (Whitehead & Whitehead, 2020). Recruitment occurred via email through the research team's professional networks, and the survey was advertised on the Occupational Therapy Australia website, Facebook, and LinkedIn, with snowball sampling encouraged (Liamputtong et al., 2017). Participants could enter a draw for a $100 AUD gift voucher by providing contact details via a separate survey administered independently of the main study to preserve anonymity.

### 2.4 Data Management and Analysis

All analyses were conducted in R (version 4.5.3; R Core Team, 2026) using base R functions and the tidyverse, ggplot2, pwr, and writexl packages. Raw data were imported from Qualtrics in CSV format with Latin‐1 encoding. Data cleaning procedures included: (a) removal of leading and trailing whitespace from all character variables; (b) conversion of empty strings to missing values (NA); (c) verification and correction of factor level ordering for categorical and ordinal variables; (d) creation of seven binary workplace context variables from comma‐separated responses to Q6; (e) integration of open‐ended text responses into categorical variables where applicable; and (f) creation of binary versions of confidence and preparedness scales for chi‐square analysis. The variables Q20 (Age of FASD clients at first contact in years) and Q21 (average duration on an FASD caseload) were excluded from all analyses due to 64.4% missing data; these variables were retained in the dataset for descriptive reporting only. The open‐ended items Q24 and Q25 were excluded from the quantitative analysis.

Binary variables were created from ordinal confidence scales to enable chi‐square testing. The binary cut‐off for confidence items (Q12, Q14_1–Q14_4, Q15) was set at ‘Confident’ OR ‘Very Confident’ versus all other responses. For the preparedness scale (Q16), the cut‐off was ‘Prepared’ OR ‘Very Prepared’ versus ‘Not Prepared’. For Q13 (ease of sourcing information), responses were dichotomised as ‘Yes’ versus ‘No/Unsure’. These definitions aligned with the conceptual meaning of confidence and preparedness and facilitated the interpretation of 2 × 2 contingency tables. For Q14_1–Q14_4, ‘Not relevant’ responses were recoded to missing prior to binary creation. All analyses retained the original ordinal variables for descriptive statistics to preserve information about the distribution of responses.

Sample size was determined by a priori using power analysis. Parameters specified an effect size of w = 0.30 (Cramér's V; medium effect), significance level α = 0.05, target power = 0.80, and degrees of freedom = 1 for 2 × 2 contingency tables. Using the pwr.chisq.test function, the required minimum sample size was N = 87. The achieved sample of N = 90 exceeded this threshold and provides adequate power to detect medium‐sized effects at the specified significance level.

Descriptive statistics comprised frequencies and percentages for all variables, reported in tables organised by descending frequency. For ordinal variables (confidence scales and resource access), the full distribution of response categories is reported alongside binary summaries to show the collapsing process.

Inferential analyses employed chi‐square tests of independence (χ^2^) for all associations among binary variables. Effect sizes were estimated using Cramér's V, interpreted as small (V ≥ 0.10), medium (V ≥ 0.30), or large (V ≥ 0.50) following Cohen's conventions. Statistical significance was set at α = 0.05.

Two primary research questions were specified a priori: (1) Is confidence in recognising FASD associated with PD attendance? and (2) Is preparedness to discuss FASD concerns associated with confidence in having reliable sources of information? Results for primary research questions are reported with Holm‐Bonferroni correction applied across the two primary tests. All remaining associations among the 12 binary variables (66 possible pairs) were exploratory, conducted post hoc, and tested with Holm‐Bonferroni correction across all 66 pairs to maintain family‐wise error control at α = 0.05. Exploratory results are clearly labelled as such in all tables and figures. Both uncorrected and Holm‐corrected P‐values are reported in summary tables to allow readers to evaluate the impact of multiple comparison correction.

Inductive content analysis (ICA) was used to identify coding schemes in the two open‐ended responses in the practice section of the survey (Vears & Gillam, 2022). This process was completed by two researchers (C. H. and A. H.) who independently analysed the data. This process involved a preliminary overall review of responses and the inductive creation of big‐picture codes. This was then followed by a second round of coding and the establishment of fine‐grained subcategories. The researchers then convened to compare coding schemas. Through discussion, they established a coding schema table. This was completed to ensure the adequacy and quality of the analysis (Braun & Clarke, 2022). The resulting table was then presented to all members of the research team and agreed upon.

### 2.5 Positionality Statement of Authors

All members of the research team possess advanced academic qualifications, a minimum of an honours degree. Three members have established backgrounds in occupational therapy and experience in both qualitative and quantitative research. Author Catherine Hilly is undertaking her PhD in FASD and occupational therapy. She delivers occupational therapy services and has engaged with family members and individuals with FASD to understand their research priorities. This has included having a FASD‐informed workforce in Australia. Collaboration within the team has been integral to their approach for comprehensive project development and data understanding.

## 3 RESULTS

### 3.1 Demographic Characteristics

Participants were predominantly female (n = 81, 91.0% of 89 respondents), with small proportions identifying as male (n = 6, 6.7%) or preferring not to disclose gender (n = 2, 2.2%). Years of occupational therapy practice varied widely: 43.3% (n = 39) had practised for 0–5 years, 15.6% (n = 14) for 6–10 years, 11.1% (n = 10) for 11–15 years, 11.1% (n = 10) for 16–20 years, and 18.9% (n = 17) for over 20 years. Most participants (94.4% of 89 respondents, n = 84) had completed their occupational therapy training in Australia; five participants trained internationally. Regarding workplace context, 65.6% (n = 59) worked in independent or private practice, 14.4% (n = 13) in not‐for‐profit organisations, and 12.2% (n = 11) in state health services. A smaller proportion worked in education settings: tertiary (7.8%, n = 7), primary (5.6%, n = 5), and secondary (4.4%, n = 4). One participant (1.1%) worked in an Aboriginal Community Controlled Health Organisation. Demographic characteristics are summarised in Table 1. Note that percentages for workplace context exceed 100% because participants could select multiple work settings.

**TABLE 1:** Demographic characteristics of occupational therapist sample (N = 90).

Characteristic	n (% of respondents)
Gender	
Female	81 (91.0)
Male	6 (6.7)
Prefer not to say	2 (2.2)
Years of OT practice	
0–5 years	39 (43.3)
6–10 years	14 (15.6)
11–15 years	10 (11.1)
16–20 years	10 (11.1)
Over 20 years	17 (18.9)
Country of occupational therapy training	
Australia	84 (94.4)
India	2 (2.2)
Canada	1 (1.1)
South Africa	1 (1.1)
Zimbabwe	1 (1.1)
Workplace context	
Independent/private practice	59 (65.6)
Not‐for‐profit organisation	13 (14.4)
State health services	11 (12.2)
Education—tertiary	7 (7.8)
Education—primary	5 (5.6)
Education—secondary	4 (4.4)
ACCHO	1 (1.1)

*Note*: Percentages for gender (n = 89 respondents) and country of training (n = 89 respondents) are calculated from those who responded to these items. Percentages for workplace context exceed 100% because participants could select multiple settings. All 90 participants provided informed consent prior to survey completion.

Abbreviation: ACCHO = Aboriginal Community Controlled Health Organisation.

### 3.2 Knowledge and Awareness

Knowledge and awareness items revealed strong agreement but inconsistent access to information. Of the 90 participants, 93.3% (n = 84) agreed that it is important to have knowledge of FASD in occupational therapy practice, whereas 6.7% (n = 6) disagreed. When asked whether they had sought further information about FASD, 74.4% (n = 67) reported having done so, versus 25.6% (n = 23) who had not. However, when asked about the ease of sourcing reliable information, only 37.8% (n = 34) reported finding it easy, 15.6% (n = 14) found it difficult, and 46.7% (n = 42) were unsure or had not yet tried. Regarding clinical experience, 65.6% (n = 59) reported having supported at least one individual with a diagnosis of FASD, whereas 34.4% (n = 31) had not. Of those with experience, 23.3% (n = 21) had referred a client for FASD screening, 56.7% (n = 51) suspected they had clients with FASD on their current caseload, whereas 23.3% (n = 21) were unsure. PD attendance was low: Only 26.7% (n = 24) had completed formal training in FASD, whereas 73.3% (n = 66) had not, were considering it, or considered it not relevant. Table 2 presents these findings in detail.

**TABLE 2:** FASD knowledge, awareness, clinical experience, and confidence (N = 90).

Item	n (% of respondents)
Agreement that FASD knowledge is important (n = 90)	
Agree	84 (93.3)
Disagree	6 (6.7)
Sought further information about FASD (n = 90)	
Yes	67 (74.4)
No	23 (25.6)
Easy to source reliable information about FASD (n = 90)	
Yes	34 (37.8)
No	14 (15.6)
Unsure/have not tried	42 (46.7)
Referred for FASD screening (n = 90)	
Yes	21 (23.3)
No	69 (76.7)
Supported individual with FASD (n = 90)	
Yes	59 (65.6)
No	31 (34.4)
Suspect FASD in current caseload (n = 90)	
Yes	51 (56.7)
No	18 (20.0)
Unsure	21 (23.3)
Completed professional development in FASD (n = 90)	
Yes	24 (26.7)
Considering	35 (38.9)
Have not considered	21 (23.3)
Not relevant	10 (11.1)
Confidence in reliable sources (n = 90)	
Not confident	30 (33.3)
Somewhat confident	49 (54.4)
Confident	11 (12.2)
Confidence recognising FASD (n = 90)	
Not confident	27 (30.0)
Neutral	39 (43.3)
Confident	21 (23.3)
Very confident	3 (3.3)
Preparedness to discuss FASD concerns (n = 90)	
Not prepared	60 (66.7)
Prepared	24 (26.7)
Very prepared	6 (6.7)
Confidence working with children with FASD (n = 89)	
Not confident	16 (18.0)
Neutral	36 (40.4)
Unsure	2 (2.2)
Confident	29 (32.6)
Very confident	6 (6.7)
Confidence working with adolescents with FASD (n = 87)	
Not confident	27 (31.0)
Neutral	30 (34.5)
Unsure	4 (4.6)
Confident	19 (21.8)
Very confident	7 (8.0)
Confidence working with adults with FASD (n = 78)	
Not confident	39 (50.0)
Neutral	17 (21.8)
Unsure	4 (5.1)
Confident	13 (16.7)
Very confident	5 (6.4)
Confidence working with families affected by FASD (n = 88)	
Not confident	28 (31.8)
Neutral	30 (34.1)
Unsure	4 (4.5)
Confident	23 (25.6)
Very confident	3 (3.4)

*Note*: Occupational therapists reported generally low to moderate confidence across Fetal Alcohol Spectrum Disorder (FASD)‐related domains. Percentages are calculated from respondents to each item. Missing data varies by item. Percentages may not sum to 100% due to rounding.

### 3.3 Confidence and Preparedness

Confidence levels regarding FASD were modest to low across all domains. When asked about confidence in having reliable sources of research‐based information about FASD, 33.3% (n = 30) reported not being confident, 54.4% (n = 49) reported being somewhat confident, and only 12.2% (n = 11) reported being confident. Regarding confidence in recognising FASD, 30.0% (n = 27) reported not being confident, 43.3% (n = 39) reported being neutral, 23.3% (n = 21) reported being confident, and only 3.3% (n = 3) reported being very confident. Preparedness to discuss FASD concerns with clients was particularly low: 66.7% (n = 60) reported not being prepared, 26.7% (n = 24) reported being prepared, and only 6.7% (n = 6) reported being very prepared.

Age‐group‐specific confidence showed variable, similar patterns across developmental groups. Confidence working with children with FASD: 18.0% (n = 16) not confident, 40.4% (n = 36) neutral, 2.2% (n = 2) unsure, 32.6% (n = 29) confident, and 6.7% (n = 6) very confident (n = 89 respondents, 1 missing). Confidence working with adolescents: 31.0% (n = 27) not confident, 34.5% (n = 30) neutral, 4.6% (n = 4) unsure, 21.8% (n = 19) confident, and 8.0% (n = 7) very confident (n = 87 respondents, 3 missing). Confidence working with adults: 50.0% (n = 39) not confident, 21.8% (n = 17) neutral, 5.1% (n = 4) unsure, 16.7% (n = 13) confident, and 6.4% (n = 5) very confident (n = 78 respondents, 12 missing). Confidence working with families: 31.8% (n = 28) not confident, 34.1% (n = 30) neutral, 4.5% (n = 4) unsure, 26.1% (n = 23) confident, and 3.4% (n = 3) very confident (n = 88 respondents, 2 missing).

### 3.4 Resource Awareness and Utilisation

Resource awareness and utilisation were generally low. The most accessed resources were the National Organisation of FASD website (42.9%, n = 36), FASD Hub website (39.5%, n = 32), and Telethon Kids Institute website (38.6%, n = 32). The University of Western Australia (UWA) certification course was currently accessed by only 3.7% (n = 3) and the Australian Foundations online course by only 22.6% (n = 19). However, many participants expressed willingness to use resources they had not yet encountered, with the Australian Foundations online course showing the highest willingness to use (52.4%, n = 44), followed by the UWA certification course (41.5%, n = 34). This is summarised in Table 3.

**TABLE 3:** FASD resource awareness and utilisation (N = 90).

Resource	Currently accessing	Willing to use	Never heard
	n (%)	n (%)	n (%)
Australian National FASD diagnostic guide (n = 84)	14 (16.7)	23 (27.4)	47 (56.0)
FASD Hub website (n = 81)	32 (39.5)	24 (29.6)	25 (30.9)
National Organisation of FASD website (n = 84)	36 (42.9)	24 (28.6)	24 (28.6)
Telethon Kids Institute website (n = 83)	32 (38.6)	22 (26.5)	29 (34.9)
Occupational therapy association training (n = 83)	15 (18.1)	29 (34.9)	39 (47.0)
Learning with FASD website (n = 83)	8 (9.6)	23 (27.7)	52 (62.7)
FARE website (n = 81)	22 (27.2)	25 (30.9)	34 (42.0)
University of Western Australia FASD certification course (n = 82)	3 (3.7)	34 (41.5)	45 (54.9)
Australian Foundations in FASD online course (n = 84)	19 (22.6)	44 (52.4)	21 (25.0)

*Note*: Response categories were collapsed for clarity: ‘Currently Accessing’ combines ‘Access Occasionally’ and ‘Accessed Regularly’; ‘Willing to Use’ combines ‘No But I Will Use it Now’ and ‘Never Used’; ‘Never Heard’ represents ‘Never Heard of This’. Percentages represent proportions of respondents to each item. Resource items had variable response rates due to differential missing data.

Abbreviation: FARE = Fetal Alcohol Research & Education.

### 3.5 Primary Analyses: Research Questions 1 and 2

#### 3.5.1 Research Question 1: Professional Development and Confidence Recognising FASD

**FIGURE 1:**
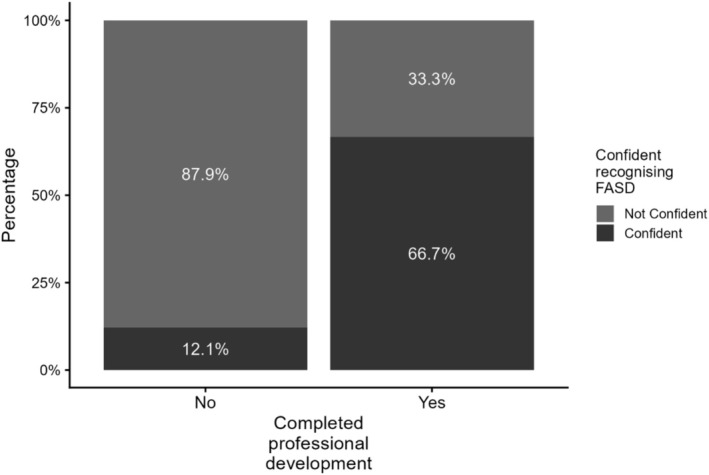
Professional development × confidence recognising FASD. *Note*: Stacked proportional bar chart showing the association between professional development completion and confidence in recognising Fetal Alcohol Spectrum Disorder (FASD). Occupational therapists who completed professional development in FASD reported substantially higher confidence in recognising FASD (66.7%) compared to those without professional development (12.1%), χ^2^(1, N = 90) = 24.06, P < 0.001, V = 0.517.

Professional development completion was significantly associated with confidence in recognising FASD, χ^2^(1, N = 90) = 24.06, P < 0.001, V = 0.517. Among occupational therapists who had completed PD in FASD (n = 24), 66.7% (n = 16) reported confidence in recognising FASD. In contrast, among those without professional development (n = 66), only 12.1% (n = 8) reported confidence. This 54.6 percentage point difference represents a large effect size (V = 0.517) and indicates that professional development substantially enhances occupational therapists' confidence in recognising FASD. This association is illustrated in Figure 1.

#### 3.5.2 Research Question 2: Preparedness to Discuss FASD and Confidence in Reliable Sources

Preparedness to discuss FASD concerns was not significantly associated with confidence in having reliable sources of information, χ^2^(1, N = 90) = 1.566, P = 0.211, V = 0.132. Among those confident in reliable sources (n = 11), 54.5% (n = 6) reported preparedness to discuss FASD. Among those not confident in reliable sources (n = 30), 16.7% (n = 5) reported preparedness. Although the observed proportions differ substantially (37.8 percentage points), this difference was not statistically significant after accounting for sampling variability. The effect size was small (V = 0.132). A warning regarding chi‐squared approximation reliability was noted due to low expected cell frequency in the ‘Not Confident & Prepared’ cell.

### 3.6 Exploratory Analyses: Professional Development and Clinical Experience

**FIGURE 2:**
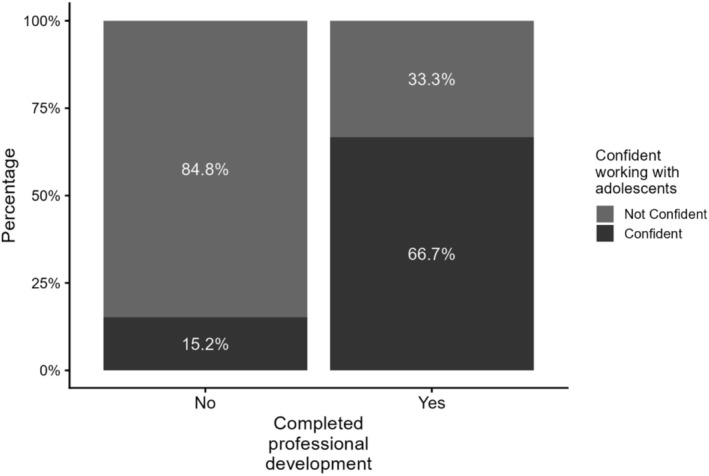
Professional development × confidence with adolescents. *Note*: Stacked proportional bar chart showing the association between professional development completion and confidence working with adolescents with FASD. Among occupational therapists who completed professional development, 66.7% reported confidence working with adolescents, compared to 15.2% without professional development, χ^2^(1, N = 90) = 20.30, P < 0.001, V = 0.475.

Beyond the primary analyses, professional development showed significant associations with confidence working with adolescents (χ^2^ = 20.30, P < 0.001, V = 0.475), illustrated in Figure 2. Among occupational therapists with professional development (n = 24), 66.7% reported confidence working with adolescents with FASD, compared to only 15.2% (n = 10) of those without training, a difference of 51.5 percentage points. This medium‐to‐large effect (V = 0.475) demonstrates the substantial value of professional development in building confidence across age groups.

**FIGURE 3:**
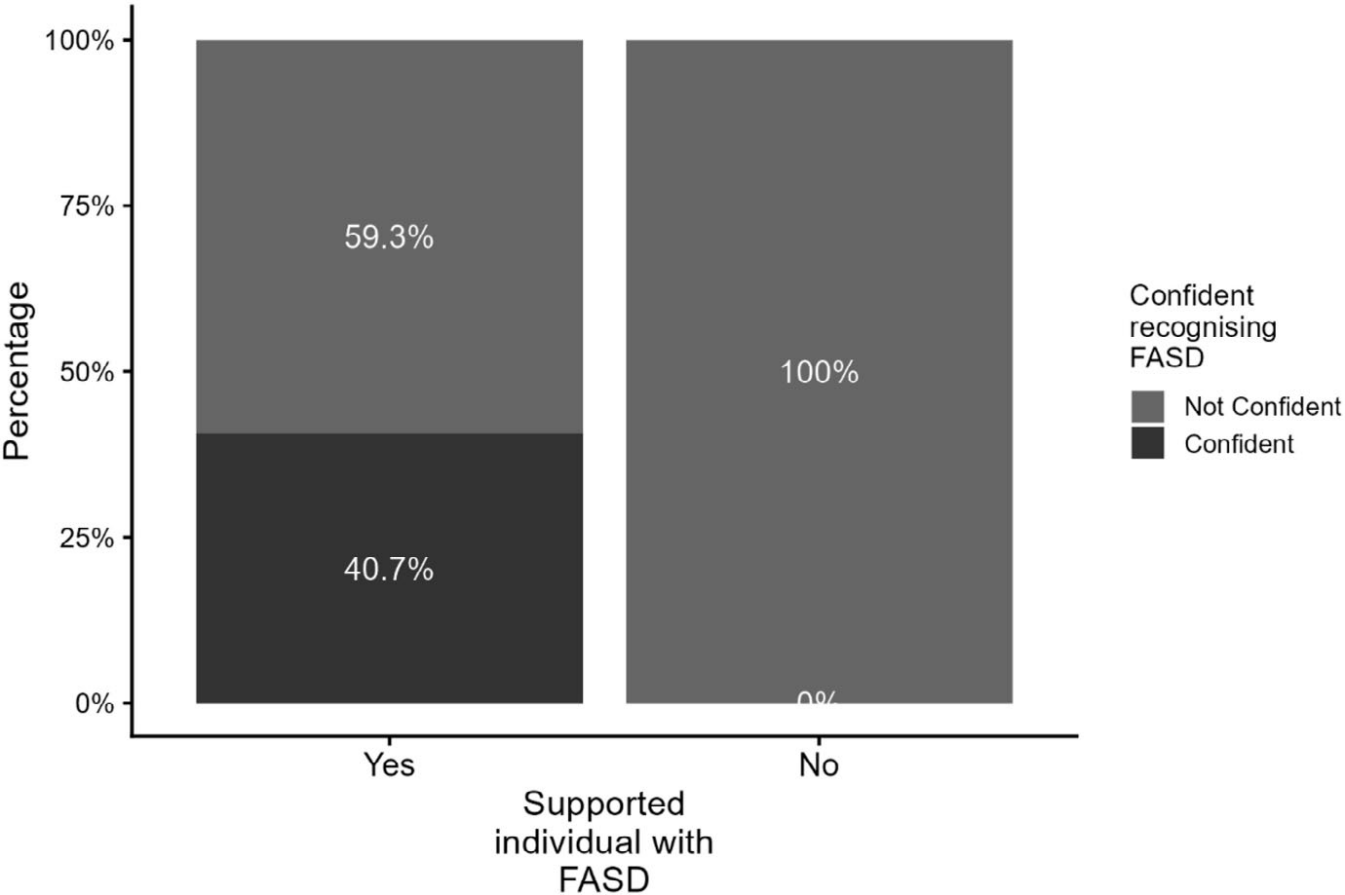
Clinical experience × confidence recognising FASD. *Note*: Stacked proportional bar chart showing the association between clinical experience supporting individuals with FASD and confidence in recognising FASD. Among occupational therapists with prior FASD experience, 40.7% reported confidence in recognising FASD, compared to 0.0% without such experience, χ^2^(1, N = 90) = 15.18, P = 0.005, V = 0.411.

Clinical experience supporting individuals with FASD was strongly associated with confidence in recognising FASD (χ^2^ = 15.18, P = 0.005, V = 0.411), illustrated in Figure 3. Among occupational therapists with prior FASD experience (n = 59), 40.7% reported confidence in recognising FASD. Notably, none of the 31 occupational therapists without FASD experience reported confidence in this domain (0.0%). This categorical threshold effect, with a 40.7 percentage point difference, suggests that clinical exposure is a critical requirement for developing confidence in FASD recognition.

### 3.7 Access to Reliable Information and Preparedness

**FIGURE 4:**
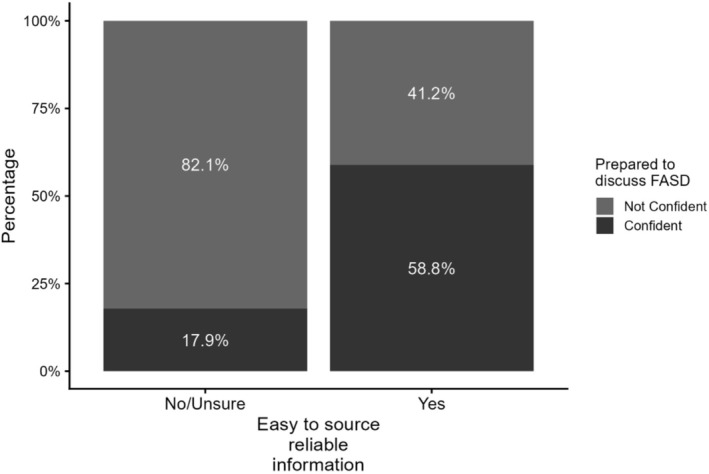
Easy to source information × preparedness to discuss FASD. *Note*: Stacked proportional bar chart showing the association between ease of accessing reliable FASD information and preparedness to discuss FASD concerns with clients. Among occupational therapists reporting easy access to reliable information, 58.8% reported preparedness to discuss FASD, compared to 17.9% who reported difficulty sourcing information, χ^2^(1, N = 90) = 14.19, P = 0.008, V = 0.397.

Access to reliable information about FASD was significantly associated with preparedness to discuss FASD concerns (χ^2^ = 14.19, P = 0.008, V = 0.397), illustrated in Figure 4. Among occupational therapists who reported easy access to reliable information (n = 34), 58.8% reported being prepared to discuss FASD with clients. Among those who reported difficulty sourcing information or had not yet tried (n = 56), only 17.9% (n = 10) reported preparedness—a difference of 40.9 percentage points. This medium effect (V = 0.397) underscores the importance of accessible resources in building clinical preparedness.

### 3.8 Exploratory Associations Among All Binary Variables

Chi‐square analysis of all 66 possible associations among 12 binary variables identified 25 significant associations after Holm‐Bonferroni correction (P < 0.05). The strongest associations were among age‐group‐specific confidence items: confidence working with children × confidence with adolescents (V = 0.724), confidence with adolescents × confidence with families (V = 0.703), and confidence with children × confidence with families (V = 0.673). These very large effects suggest that confidence across different age groups is highly interdependent, potentially reflecting an underlying general construct of FASD‐specific confidence. Additional exploratory findings are consistent with primary findings, showing significant associations between professional development, clinical experience, information access, and confidence across multiple domains.

### 3.9 Summary of Key Quantitative Findings

Professional development in FASD emerges as the most robust predictor of occupational therapists' confidence across multiple domains. Occupational therapists who completed formal training demonstrated substantially higher confidence in recognising FASD (66.7% vs. 12.1%), working with adolescents (66.7% vs. 15.2%), and working with children (65.0% vs. 10.6%) compared to those without training. Clinical experience supporting individuals with FASD was strongly associated with confidence in recognising FASD (40.7% vs. 0.0%), with no participant without prior FASD experience reporting confidence in this domain. This categorical threshold effect suggests that clinical exposure is a critical requirement for developing confidence in FASD recognition. Access to reliable information about FASD was an important facilitator of preparedness to discuss FASD concerns with clients (58.8% vs. 17.9%).

These findings collectively underscore the importance of professional development, the critical value of clinical experience, and the need to improve the accessibility of trustworthy FASD resources for occupational therapists. Despite broad agreement (93.3%) that knowledge of FASD is important in occupational therapy practice, many participants reported low confidence and preparedness, indicating a significant knowledge–practice gap in FASD recognition and management among occupational therapists in Australia.

### 3.10 Inductive Content Analysis

There were 49 responses to the two open‐ended questions. When exploring how occupational therapists guide their approach when working with individuals with FASD and their families, qualitative findings suggested that in the absence of occupational therapy–specific FASD guidance, respondents relied on general occupational therapy theory, clinical experience, and comparative knowledge from other diagnoses to inform their practice.

Four coding schemes were identified following inductive content analysis: (i) occupational therapy theory‐driven practice, (ii) practice experience with FASD and other diagnoses, (iii) FASD knowledge and resources, and (iv) lack of FASD awareness (Table 4). These coding schemes encompassed the use of goals, models and frames of reference, professional reasoning, lived and comparative experience, and engagement with research, online resources, and professional development. This reliance on general occupational therapy frameworks and comparative clinical experience is consistent with the quantitative finding that only 26.7% of respondents had completed formal FASD‐specific PD. These responses provide insights into how occupational therapists are currently practising to support individuals with FASD and their families.

**TABLE 4:** Coding schemes and example responses.

What do you use to guide your choice of approach when working with individuals with FASD and their family?		
Coding schema	Sub schema	Example response
Occupational therapy theory driven practice	Goals	‘COPM for goal setting, GAS goal format for outcome Measure’.
		‘GAS goalsetting’.
	Type of approach	‘Family centred approach. My experience has shown there are generally more difficulties within the family unit for children with FASD. Such as parents with executive function/developmental difficulties themselves or other siblings with developmental difficulties’.
		‘We use a parent/carer coaching informed model for all clients, based on F‐Words principles (i.e. functional, participation‐based goals rather than clinician led goal setting). Intervention trends towards a combination of goal‐directed training and carer/educator focused co regulation supports’.
	Models and frames of reference	‘PEOP, MOHO, SEWB depending on the goals required’.
		‘Biomechanical and behavioural approaches’.
		‘COPM, PRPP, OPMA’.
Practice experience with FASD and other diagnoses	Professional reasoning	‘Dependent on the situation. With children, keyworker model, strengths‐based approaches, trauma‐informed practice, neurodevelopmental approaches, positive behaviour support, sensory regulation. With adults, a person‐centred approach, positive behaviour support, quality of life framework, co‐production’.
		‘Professional reasoning, knowledge of neurocognitive impairment with FASD, hearing from the living experiences of people with FASD and their families’.
	Lived experience	‘Hearing from the living experiences of people with FASD and their families’.
	Comparative knowledge	‘Similar approach to supporting emotional regulation and executive functioning deficits for ADHD and ASD participants’.
		‘Similar approach to children with lower IQ or finding topics challenging. Taking tasks slowly and reframing if needed. Using more hands‐on approach and concrete items has helped’.
FASD knowledge and resources	Research	‘The Novak paper ‐ top‐down/goal directed approaches with caregiver education’.
		‘Australian Guide to the diagnosis of FASD Diagnostic Guide’
	Free/online	‘FASD resources have informed much of [my] input’.
		‘FASD website’.
		‘FASD websites (international) more around parent education’.
	Conferences and professional development	‘I have in the past been provided with information from international conferences and I often annually search on latest updates research best practices’.
Lack of FASD awareness		‘Not sure I incorporate my experience ‐ tend not rely on occupational therapy information as occupational therapy is also slow to the party’.

What further knowledge/resources would you like to guide your practice with individuals with FASD and their families?		
Coding schema	Sub schema	Example response
Occupational therapy practice	What resources can occupation therapists use?	‘Literally anything, there's not much information available’.
		‘Are there things I should be doing differently for FASD?’
		‘If I were to see children with FASD now I would engage in more specific PD training, to ensure that I was up to date with the state of the evidence to inform more therapeutically specific intervention’.
		‘Websites you've mentioned will be helpful. I am not familiar with them all’.
Knowledge for occupational therapists to support clients	Supporting families and people with FASD	‘Assessment service but no ongoing therapy avenues (not funded by NDIS for FASD alone)’.
		‘Local community support systems including a list of local schools that provide support to individuals with FASD. Names of local paediatricians who can diagnose FASD’.
		‘There isn't much information I gain being a Sydney based practitioner. Despite Westmead having an assessment department I really struggled in the past even just to get families referred to this service’.
Knowledge about FASD‐specific occupational therapy practice	Knowledge about occupational therapy practice with FASD and learning specific to FASD	‘How to support families exploring a diagnosis, being confident in advocating concerns about possible FASD as well as information about the process of diagnosis’.
		‘I'm currently completing a graduate certificate. I would like more support regarding specific strategies to use in sessions’.
		‘More occupational therapy relevant training not just around diagnosis’.
		‘Occupational therapy specific guides’.
	Knowledge about FASD	‘Presentation features and evidence‐based interventions. Info on trajectory’.
		‘Training in recognising this more easily, decreasing shame, faster referral for formal diagnosis’.

Three coding schemes were identified when analysing ‘What further knowledge/resources would you like to guide your practice with individuals with FASD and their families?’ (Table 4): (i) occupational therapy practice resources, (ii) knowledge to support occupational therapists in working with individuals with FASD and their families, and (iii) knowledge about FASD and FASD‐specific occupational therapy practice, including assessment, intervention strategies, and diagnostic processes. These responses suggest that occupational therapists desired occupational therapy–specific resources and guidance to better support individuals with FASD and their families, given that such resources are currently not readily available. This desire for additional resources and guidance aligns with the quantitative finding that only 37.8% of respondents reported easy access to reliable FASD information and that easy access was significantly associated with greater preparedness to discuss FASD concerns with clients (58.8% vs. 17.9%, P = 0.008). These responses highlight the additional resources and knowledge that Australian occupational therapists desire to enhance their practice. This is summarised in Table 4.

## 4 DISCUSSION

This study explored occupational therapists' awareness, knowledge, and practices relating to FASD within an Australian context and their desire for additional training and resources to support practice. Suggestions to enhance FASD‐informed occupational therapy practice are provided.

### 4.1 Occupational therapy FASD awareness and scope of practice

Referral for FASD assessment when PAE is reported in a client's history is one component of occupational therapy practice with individuals suspected of FASD. As FASD presentation can vary and evidence‐based, occupation‐focussed interventions remain limited (Hilly et al., 2024), occupational therapists rely on awareness, clinical reasoning, theoretical models, and experience to guide practice. Recognising FASD is important to ensure individuals with FASD or suspected FASD and their families receive effective support. As individuals may present to occupational therapists prior to diagnosis due to occupational performance challenges, occupational therapists have a role in advocating for and screening for FASD by exploring PAE in prenatal history gathering (Australian Guidelines Development Group, 2024). Our results indicate that most respondents did not feel confident recognising FASD, nor prepared to discuss referral for potential diagnosis with clients or families—presenting a challenge to early detection and appropriate assessment.

### 4.2 FASD knowledge‐seeking behaviours of Australian occupational therapists

Survey results indicated that occupational therapists' FASD awareness plays an important role in their knowledge‐seeking behaviour. Two streams of initial exposure to FASD were identified: (i) clinical experience through client contact and (ii) formal instruction within the university curriculum.

Early engagement with FASD education within the occupational therapy curriculum may promote a FASD‐prepared workforce. Maclean et al. (2014) found that occupational therapy students, within a Scottish university, considered FASD knowledge important to their role, a finding echoed by nearly all respondents in our study. We did not investigate prior FASD training, so the impact of pre‐existing knowledge on respondents' views cannot be determined.

With updated Australian diagnostic guidelines (Australian Guidelines Development Group, 2024), recognition of FASD under Medicare, and NDIS inclusion, growing numbers of individuals are likely to be screened and diagnosed with FASD in Australia. It is therefore unsurprising that most respondents reported first becoming aware of FASD through client contact.

Respondents most commonly reported first seeing clients with FASD between ages four and seven. Early diagnosis provides a valuable opportunity for tailored intervention during critical developmental periods (Dawe et al., 2023; Skorka et al., 2020), and occupational therapy involvement at this stage is key to reducing costs associated with missed diagnoses (Jirikowic et al., 2008; McDougall et al., 2020). A small number of respondents reported working with adults with FASD, and respondents reported the least confidence in this age group.

For occupational therapists encountering FASD for the first time, some drew on FASD‐specific resources to address knowledge gaps, whereas others did not know where to access these or had not sought them out. A recent scoping review conducted by Stubbs et al. (2024) evaluated 41 resources aimed to aid health professionals with referral and support for people with FASD using the Appraisal of Guides for Research and Evaluation II instrument and an adapted version of the National Health and Medical Research Council FORM Framework and iCAHE Guideline Quality Checklist. Most of the resources were published in the United States and Australia and scored between 76% and 100% on an overall quality assessment and were recommended for use in an Australian context. The authors reported that most of the guides and factsheets (22/32) were rated as ‘good’. Our findings similarly indicated that occupational therapists with easy access to reliable information were significantly more likely to feel prepared to discuss FASD with clients (58.8% vs. 17.9%, P = 0.008). However, confidence in reliable sources alone did not reach statistical significance (P = 0.211), suggesting ease of access may be a more important facilitator of preparedness than source confidence per se. Although there is a wide range of resources available, our results suggest that occupational therapy‐focussed evidence‐based information is required, with many therapists seeing individuals with FASD and their families reporting a lack of occupational therapy‐specific practice knowledge. This finding is supported by research, which has also found a lack of evidence‐based and participation‐focussed practices among occupational therapists (Hilly et al., 2024; Skorka et al., 2022). Limited FASD knowledge has been shown to increase burden on individuals and caregivers seeking support (Chamberlain et al., 2017). One caregiver specifically referenced occupational therapy in describing this gap: ‘We went to occupational therapy for a bit and they said, ‘she'll be right, she'll be right.’ But things were just not right (P4)’ (Chamberlain et al., 2017, p. 103).

Respondents felt unprepared to raise concerns about suspected FASD or refer for screening. Evans et al. (2024) found that therapists without FASD training in the past 5 years reported significantly lower referral confidence. Our respondents similarly reported that engagement with FASD resources and professional development (PD) increased confidence in recognising FASD‐associated deficits—consistent with Brimacombe et al. (2008) and Chamberlain et al. (2017) and with broader international literature (Birch et al., 2016; Chu et al., 2023; McLachlan et al., 2021; Rudeen et al., 2007). Engagement in FASD‐related PD varied across respondents, potentially due to caseload composition, stigma, limited awareness, or other factors. Most respondents had supported individuals with FASD for less than 5 years and had not considered FASD‐specific PD. Data on caseload duration were excluded from formal analysis due to high rates of missing data (64.4%), so conclusions about its relationship to PD engagement cannot be drawn. Factors influencing PD choice include availability, cost, organisational support, perceived importance, and competence (Pang et al., 2024). Personal biases may also prevent clinicians from recognising the value of FASD‐specific PD; this warrants further examination.

Importance of context (ICA) analysis suggested two approaches to FASD‐specific PD may be beneficial: (i) tailored occupational therapy knowledge and practice‐specific learning and (ii) guidance on accessing universal FASD knowledge. Respondents sought evidence‐based, occupation‐focussed interventions and identified a need for education on FASD presentation and differential diagnosis—for example, one participant noted wanting information about ‘diagnostic differences between FASD and ASD during diagnosis’.

Occupational therapists with experience supporting individuals with FASD expressed a desire for more information and access to services. Evans et al. (2024) identified similar challenges, including lengthy wait times affecting motivation to engage in FASD care planning. Managing the diverse range of professionals involved remains a significant challenge for both practitioners and families (Bagley, 2019). As FASD complexity and co‐occurring diagnoses increase across the lifespan (Vanderpeet, 2025), individuals with FASD and their families will encounter occupational therapists across a range of clinical settings. Our findings indicate that whereas some occupational therapists actively seek FASD‐specific knowledge from sources such as NOFASD Australia (n.d.) and international websites, others do not seek additional information at all.

### 4.3 Occupational therapy practice

Our survey respondents reported using occupational therapy frameworks, frames of reference, and theory‐driven practices to support individuals with FASD and their families. To address occupational therapy goals, they often rely on clinical experience or comparative knowledge of other diagnoses, such as autism, to inform their choice of intervention. However, the complexity of the presentation of individuals with FASD, which often increases across the life span, requires FASD‐specific knowledge and approaches (Chamberlain et al., 2017; Dawe et al., 2023; Evans et al., 2024; McLachlan et al., 2021; Vanderpeet, 2025). In a survey study exploring Australian occupational therapists' practice with children with Developmental Coordination Disorder (DCD), Hunt et al. (2026) described the habitual use of practice frameworks by occupational therapists when working with children with DCD, when there was an absence of diagnosis‐specific frameworks. They reported a lack of awareness of Australian occupational therapists regarding the distinct needs of children with DCD. Likewise, our results indicated that some occupational therapists displayed a similar lack of awareness of FASD due to an absence of FASD‐specific frameworks for occupational therapists and were also reliant on more general occupational therapy practice reasoning models. This was further supported by qualitative findings, in which theory‐driven occupational therapy practice and comparative clinical experience with other diagnoses emerged as the predominant approaches used by respondents to support individuals with FASD, highlighting the absence of FASD‐specific guidance to inform their practice.

## 5 LIMITATIONS OF THIS STUDY

This study has several limitations. First, respondents were not asked what percentage of their caseload involved clients with FASD or the age range of their caseload, so we could not compare their responses to their total caseload. Unlike other studies, we did not directly ask respondents about FASD‐specific knowledge, as these studies have focussed on perceived knowledge and therefore could not compare their responses with ours (Brimacombe et al., 2008; Chu et al., 2023; Kerimofski et al., 2024; Rudeen et al., 2007). Instead, we chose to explore where and how occupational therapists acquired knowledge about FASD and identify their knowledge gaps. The survey was open for only 8 weeks, which may have limited opportunities for participation, as some respondents may not have had enough time to complete the survey, or potential participants may not have been reached. However, the achieved sample size exceeded the minimum threshold determined by a priori power analysis, providing adequate power to support meaningful interpretation of the findings. The survey was piloted and refined based on feedback from occupational therapists working with the FASD community; however, future research should more explicitly report and strengthen engagement with the Australian occupational therapy and FASD community in the conception, design, implementation, and intended translation of the survey, with limited co‐design representing a potential limitation.

Although this study had limitations, it had strengths. The study was the first Australian research to focus on occupational therapists' knowledge, awareness, and practices regarding FASD. It was intended to capture broad information from occupational therapists and is being followed by a subsequent study using in‐depth interviews with occupational therapists who work with children and adolescents with FASD to explore their practice. The results demonstrated representation across most Australian states and territories with respondents from both metropolitan and rural Modified Monash categories (Australian Government, 2023).

### 5.1 Implications for occupational therapy practice

Many occupational therapists in our study reported using occupational performance approaches and models when working with individuals with FASD and their families. However, their approach is often not adjusted to the specific neurocognitive profile and needs of this population. Results indicate that most occupational therapists in our study sought more practice and research guidance in occupational participation to support individuals with FASD across the lifespan and their families. FASD‐specific guidance with practical approaches may assist occupational therapists who often apply non‐FASD‐informed evidence and practices in the absence of such guidance (McLachlan et al., 2021; Rudeen et al., 2007).

Our research suggests that enhancing occupational therapists' knowledge of FASD and providing them with FASD‐specific occupational therapy resources is likely to foster a more informed occupational therapy workforce. This, in turn, may lead to improved client outcomes for individuals with FASD. Currently, occupational therapists employ occupational performance models and approaches when supporting individuals with FASD and their families. However, these approaches often lack adjustments specific to the unique neurocognitive profiles and lifelong challenges of individuals with FASD. Our findings align with international research indicating a critical gap in occupational therapists' access to practical, FASD‐specific guidance, and evidence‐based strategies to support occupational participation across the lifespan. Without such tailored resources, occupational therapists may default to general approaches that are not adequately informed by the distinct characteristics of FASD (Petrenko & Alto, 2017).

## 6 CONCLUSION

Currently, there appear to be gaps in occupational therapists' knowledge and awareness of FASD in Australia. Occupational therapists in this study appear to draw on their own clinical experience and general occupational therapy frameworks to guide their approaches to supporting individuals with FASD and their families. Improved awareness of and access to existing resources, alongside occupational therapy–specific practice guidance and research, may better support occupational therapy services for individuals with FASD across the lifespan. Accessible PD and occupational therapy–specific practice resources, such as practice guidelines, intervention tools, and FASD‐specific frameworks, may contribute to a more FASD‐informed workforce. Qualitative findings further highlighted that occupational therapists desired occupational therapy–specific practice resources, including assessment tools, intervention strategies, and guidance on diagnostic processes. Our research suggests that increasing occupational therapists' knowledge and providing FASD‐specific occupational therapy resources may foster a more informed occupational therapy workforce, potentially leading to improved outcomes for individuals with FASD and their families.

## AUTHOR CONTRIBUTIONS

All authors revised and approved the final manuscript. **Alison Houghton:** development, ethical application, methodology, formal analysis of qualitative and quantitative data, writing—draft, review and editing. **Laine Chilman:** conceptualisation, ethical application, methodology, guided formal analysis of quantitative data, writing, review and editing. **Asmita Mudholkar:** development review, ethical application, methodology, writing—review and editing. **Catherine Hilly:** development review, ethical application, methodology, formal analysis of qualitative data and writing review and editing. **Daniel Meloncelli:** formal analysis of quantitative data, writing—draft, review and editing.

## CONFLICT OF INTEREST STATEMENT

The authors declare that they have no competing interests.

## ETHICS STATEMENT

Ethics approval was obtained from the Human Research Ethics Committee at The University of the Sunshine Coast (Approval S241971).

## DECLARATION

Grammarly (paid version) was used to enhance the clarity and fluency of Australian English expression and to assist with sentence refinement in this document. All authors reviewed and approved the final version of the manuscript.

## Data Availability

The data that support the findings of this study are available on request from the corresponding author. The data are not publicly available due to privacy or ethical restrictions.
